# Risk factors and myocardial infarction in patients with obstructive sleep apnea: impact of β2-adrenergic receptor polymorphisms

**DOI:** 10.1186/1741-7015-5-1

**Published:** 2007-01-01

**Authors:** Nina K Bartels, Jan Börgel, Stefan Wieczorek, Nikolaus Büchner, Christoph Hanefeld, Daniel Bulut, Andreas Mügge, Lars C Rump, Bernd M Sanner, Jörg T Epplen

**Affiliations:** 1 Human Genetics, Ruhr-University Bochum, Germany; 2 Medical Clinic II Cardiology and Angiology, St. Josef-Hospital/Bergmannsheil, Ruhr-University Bochum, Germany; 3 Medical Clinic I, Marien-Hospital Herne, Ruhr-University Bochum, Germany; 4 Medical Clinic, Bethesda-Hospital, Wuppertal, Germany

## Abstract

**Background:**

The increased sympathetic nervous activity in patients with obstructive sleep apnea (OSA) is largely responsible for the high prevalence of arterial hypertension, and it is suggested to adversely affect triglyceride and high-density lipoprotein (HDL) cholesterol levels in these patients. The functionally relevant polymorphisms of the β*2*-adrenergic receptor (Arg-47Cys/Arg16Gly and Gln27Glu) have been shown to exert modifying effects on these risk factors in previous studies, but results are inconsistent.

**Methods:**

We investigated a group of 429 patients (55 ± 10.7 years; 361 men, 68 women) with moderate to severe obstructive sleep apnea (apnea/hypopnea index (AHI) 29.1 ± 23.1/h) and, on average, a high cardiovascular risk profile (body mass index 31.1 ± 5.6, with hypertension in 60.1%, dyslipidemia in 49.2%, and diabetes in 17.2% of patients). We typed the β*2*-adrenergic receptor polymorphisms and investigated the five most frequent haplotypes for their modifying effects on OSA-induced changes in blood pressure, heart rate, and lipid levels. The prevalence of cardiovascular risk factors and coronary heart disease (*n *= 55, 12.8%) and survived myocardial infarction (*n *= 27, 6.3%) were compared between the genotypes and haplotypes.

**Results:**

Multivariate linear/logistic regressions revealed a significant and independent (from BMI, age, sex, presence of diabetes, use of antidiabetic, lipid-lowering, and antihypertensive medication) influence of AHI on daytime systolic and diastolic blood pressure, heart rate, prevalence of hypertension, and triglyceride and HDL levels. The β*2*-adrenergic receptor genotypes and haplotypes showed no modifying effects on these relationships or on the prevalence of dyslipidemia, diabetes, and coronary heart disease, yet, for all three polymorphisms, heterozygous carriers had a significantly lower relative risk for myocardial infarction (Arg-47Cys: *n *= 195, odds ratio (OR) = 0.32, *P *= 0.012; Arg16Gly: *n *= 197, OR = 0.39, *P *= 0.031; Gln27Glu: OR = 0.37, *P *= 0.023). Carriers of the most frequent haplotype (*n *= 113) (haplotype 1; heterozygous for all three polymorphisms) showed a five-fold lower prevalence of survived myocardial infarction (OR = 0.21, *P *= 0.023).

**Conclusion:**

Our study showed no significant modifying effect of the functionally relevant β*2*-adrenergic receptor polymorphisms on OSA-induced blood pressure, heart rate, or lipid changes. Nevertheless, heterozygosity of these polymorphisms is associated with a lower prevalence of survived myocardial infarction in this group with, on average, a high cardiovascular risk profile.

## Background

Patients with obstructive sleep apnea (OSA) show increased sympathetic nervous activity during both night and day [1-3]. This increased sympathetic activity may contribute to arterial hypertension, increased heart rate [4,5], and dyslipidemia [6,7]. In addition to exacerbation of these cardiovascular risk factors, the elevated sympathetic activity makes patients with OSA vulnerable to both fatal and non-fatal coronary events [8-10]. Sympathetic activity is mediated by β*2*-adrenergic receptors, among others. The receptors are expressed in a wide variety of tissues, including the vascular and bronchial smooth muscles, the heart, and adipocytes. Thus, β*2*-adrenergic receptors play a critical role in the regulation of blood pressure (BP), heart rate, and lipid metabolism. Several polymorphisms of the β*2*-adrenergic receptor gene have been identified. For three polymorphisms, which are the subject of the present study, functional consequences have been described [11]:

1) At position -47 in the untranslated region of the β*2*-adrenergic receptor gene, a cytosine (C)/thymine (T) polymorphism results in either arginine (Arg) or cystine (Cys) in the 5' leader sequence. This sequence encodes a peptide that modifies β*2*-adrenergic receptor gene translation. In human airway smooth muscles, the Cys variant showed a consistently greater expression of the β*2*-adrenergic receptor [12,13].

2) At position 46 of the coding region, adenine (A) or guanine (G) can be found, resulting in arginine (Arg) or glycine (Gly) at amino acid position 16.

3) At nucleotide position 79, a C/G polymorphism results in glutamate (Glu) or glutamine (Gln) at amino acid position 27.

The latter two polymorphisms and their haplotypes modify the down-regulation of the β*2*-adrenergic receptor upon agonist stimulation. In a previous study [14], the Gly16/Gln27 haplotype underwent enhanced down-regulation compared with the Arg16/Gln27 haplotype, while the Arg16/Glu27 haplotype showed almost no down-regulation. Many population-based studies have been performed to investigate the modifying potency of these polymorphisms on cardiovascular risk factors *in vivo*, but the results have been inconsistent or contradictory.

Previously, a significant negative influence of the severity of OSA on BP, prevalence of arterial hypertension, heart rate, and serum levels of high-density lipoprotein cholesterol (HDL-C) was shown in the parent study cohort of the patients [4,7] included in this report. In the present study, we scrutinised the modifying effects of β*2*-adrenergic receptor polymorphisms on these risk factors, which are mediated by elevated sympathetic activity. We further investigated whether the three polymorphisms have an effect on the comparatively high prevalence of coronary heart disease (CHD) and survived myocardial infarction (MI) in our patients with OSA.

## Methods

### Patients and parent study data

The study protocol was approved by the local ethics committee (Ruhr-University Bochum, Germany). Patients were consecutively enrolled during the years 1999 to 2001 in the sleep laboratory of the Marienhospital, Herne, Ruhr-University Bochum, Germany. The patients were admitted to the hospital because of a history of apnea, snoring, or hypersomnic symptoms such as daytime tiredness or impairment of cognitive functions.

Detailed information on the determination of cardiac risk factors and polysomnography have been described extensively in previous publications [4,7]. In brief, after informed consent, the severity of OSA was assessed using the apnea/hypopnea index (AHI). Dyslipidemia was diagnosed by the presence of anamnesis and use of lipid-lowering drugs or presence of dyslipidemic serum levels. For blood analysis, the following cut-off values were defined as abnormal: total cholesterol ≥ 200 mg/dL, triglycerides ≥ 180 mg/dL, HDL-C ≤ 40 mg/dL, and low-density lipoprotein cholesterol (LDL-C) ≥ 150 mg/dL. Diabetes was defined by presence of anamnesis and use of antidiabetic medication or insulin treatment. The diagnosis of hypertension was established by use of antihypertensive medication or if the mean of three measured BP readings exceeded 140 mmHg (systolic) or 90 mmHg (diastolic).

### Genotyping

Peripheral blood samples from the patients were taken after they had given informed consent.

The following primers were synthesised to amplify a 283-bp fragment containing the Cys-47Arg polymorphism: 5'-AATGAGGCTTCCAGGCGTC-3' (sense) and 5'-ACTTGGCAATGGCTGTGATG-3' (antisense). PCR was carried out in a final volume of 10 μL with 50 ng DNA, 2 mM dNTPs and 1 U *Taq *polymerase. PCR cycling started with initial denaturation for 5 minutes at 94°C. The annealing temperature of the first cycle was 69°C, second cycle 66°C and the remaining 28 cycles 63°C, each cycle being 1 minute. Extension was performed at 72°C for 1 minute (final extension 5 minutes). Allele-specific restriction digests were performed with *MspA*II, which fails to digest the Cys-47 allele because of the C→G transition. Digested DNA was separated by electrophoresis on 1.5% agarose gels.

For the Gly16Arg polymorphism, the following primers were synthesised to obtain a 96-bp fragment: 5'-GCCAGTGCGCTTACCTGC-3' (sense) and 5'-TGGTCCGGCGCATGGCTT-3' (antisense). Final volume of the PCR was 10 μL, containing 50 ng DNA, 2 mM dNTPs, 1 U *Taq *polymerase and 1% radioactively labelled dATP. The PCR cycles after denaturation (94°C for 5 minutes) were 70°C (1 cycle), 67°C (1 cycle) and 64°C (28 cycles), with each cycle lasting 1 minute. Extension at 72°C was performed for 1 minute, and 5 minutes at the end. The PCR products were digested with the restriction endonuclease *Nco*I in order to optimize the demonstration of the polymorphism by single-strand conformational polymorphism analysis. Restriction products were separated in an 8% polyacrylamide (19:1 acrylamide:bisacrylamide) gel containing 10% glycerol.

For genotyping the Gln27Glu polymorphism, the primers 5'-AAGCCATGCGCCGGACCA-3' (sense) and 5'-ACTTGGCAATGGCTGTGATG-3' (antisense) primers were chosen, which produce a 134-bp long product. PCR was performed with a final volume of 10 μL comprising 50 ng DNA, 2 mM dNTP and 1 U *Taq *polymerase. The chosen temperatures after denaturation were 70.5°C (1 cycle), 67.5°C (1 cycle), and 64.5°C (28 cycles). Allele-specific restriction was performed with *Bbv*I and bands separated in a 1.5% agarose gel.

### Statistical analysis

Results are presented as means +/-SD. All reported *P*-values are two-tailed. Statistical analyses were performed with SPSS for Windows software (SPSS, Chicago, IL, USA). Multivariate linear regressions were calculated in order to determine independent associations between the AHI and BP, heart rate and lipid serum levels in the presence of body mass index (BMI), age, and sex. The relationship between the severity of OSA represented by the AHI and the prevalence of arterial hypertension was calculated using multiple logistic regression analysis using BMI, age, and sex as covariates. Demographic characteristics of the patients with OSA were compared between the different genotypes using the χ^2 ^test; *P *< 0.05 was considered significant. The phenotypic values were compared between the genotypes using analysis of variance (ANOVA). Student's *t*-test for unpaired samples was used for comparisons between particular genotypes/haplotypes and the rest of the group.

An additional model was established in which the homozygous states of each polymorphism were pooled in one group and compared with the heterozygous genotype. The influence of heterozygosity of each polymorphism on survived MI was then analysed in a multivariate logistic regression model, adjusted for diabetes, dyslipidemia, hypertension, sex, BMI, AHI, and age.

## Results

The prevalences of the three genotypes are shown in Table 1, which also summarises the demographic findings in the full study group (*n *= 429), separated for the different genotypes of the β*2*-adrenergic receptor polymorphisms.

Allele frequencies of the different polymorphisms were: Arg-47Cys = 0.53/0.47, Arg16Gly = 0.37/0.63 and Gln27Glu = 0.49/0.51. The β*2*-adrenergic receptor polymorphisms Arg-47Cys and Gln27Glu were found to be strongly concordant, generating the haplotypes -47Cys/27Glu (83.5% of -47Cys homozygotes are 27Glu homozygotes). The five most common haplotypes accounted for 73.2% of all observed haplotypes.

Use of antihypertensive or lipid-lowering medication was distributed equally in the genotype subgroups and consisted of angiotensin-converting enzyme inhibitors (*n *= 128; 29.8%), β-receptor blockers (*n *= 87; 20.3%), diuretics (*n *= 70; 16.3%), calcium-channel blockers (*n *= 86; 20.0%), nitrates (*n *= 41; 9.6%) and α-receptor blockers (*n *= 1; 0.2%). Of the 40 patients on lipid-lowering medication, 37 were using statins and 3 fibrates. Total cholesterol and triglycerides measurement was available for 360 patients, and lipoprotein determination for 278 patients.

The mean ± SD AHI in our study group was 29.1 ± 23.1 events per hour, and the mean minimal nocturnal oxygen saturation was 81.0 ± 10.9%. Linear and independent relationships between the AHI and daytime systolic and diastolic BP, daytime heart rate, the prevalence of hypertension, and HDL-C serum levels have been shown in this study group previously [4]. Because DNA tests could not be performed for all members of the parent study cohort (*n *= 540), we performed a new, adjusted regression for our subgroup of 429 subjects [7]. Multivariate linear or logistic regressions revealed a significant influence of the severity of OSA on daytime systolic BP (β = 0.129, *P *= 0.008), diastolic BP (0.129, *P *= 0.012), heart rate (β = 0.197. *P *< 0.001) prevalence of hypertension (β = 0.026, *P *= < 0.001), and HDL-C serum levels (*n *= 78, β = -0.146, *P *= 0.003)[7]. This influence was independent of diabetes, BMI, age, sex, or antidiabetic, lipid-lowering, or antihypertensive treatments.

As shown in Table 2, the β*2*-adrenergic receptor polymorphisms did not modulate these relationships (Table 2; hypertension is shown in Table 1).

There were no significant differences between the β*2*-adrenergic receptor genotypes in relation to BMI, AHI (not shown), medication (not shown) or the prevalence of hypertension, diabetes, dyslipidemia, and CHD. Patients heterozygous for the Gln27Glu polymorphism were significantly older than those with homozygous genotypes (Table 1).

Of the 429 patients with OSA, 27 (6.3%) had survived a MI. Patients heterozygous for the Arg-47Cys and Gln27Glu polymorphisms showed a lower rate of survived MI than those with the homozygous forms. There was a trend towards a lower rate of survived MI in patients heterozygous for the Arg16Gly polymorphism (Table 1).

Table 3 shows the cardiovascular risk factors and diseases for the five most frequent haplotypes. Except for survived MI, there were no significant differences between the haplotypes. Haplotype 1 with heterozygosity for all three polymorphisms showed a lower rate of survived MI. As shown in Table 4, patients heterozygous for all three polymorphisms had a significantly lower relative risk (RR) for MI compared with homozygous subjects. Carriers of the most frequent haplotype 1 (*n *= 113), with heterozygosity for all three polymorphisms, showed a five-fold lower prevalence of survived MI (OR = 0.21, *P *= 0.023). When the homozygous and heterozygous states of each polymorphism were pooled into groups, the multivariate logistic regression model revealed an protective influence on survived MI, independent of age, BMI, AHI, diabetes, dyslipidemia, hypertension and sex [Arg-47Cys: β = -1.23, *P *= 0.032; Arg16Gly: β = -0.94, *P *= 0.53; Gln27Glu: β = -1.12, *P *= 0.27; haplotype 1 (all heterozygous): β = -1.555, *P *= 0.04].

## Discussion

We analysed β*2*-adrenergic receptor polymorphisms in a study group of untreated patients with OSA in whom a linear and independent relationship between the frequency of nocturnal apnea/hypopnea and daytime systolic and diastolic BP, daytime heart rate, prevalence of arterial hypertension, and HDL serum levels were shown previously [4,7]. The elevated sympathetic nerve activity in patients with OSA [1-3] is suggested to mediate these effects, especially in relation to the cardiovascular system.

The underlying mechanisms between the severity of OSA and modulation of lipid serum level have been discussed in detail elsewhere [7]. The fact that we could not find any influence despite the sympathetic pathway supports the notion that the effect of these polymorphisms *in vivo *is minor, and confirms previous negative results [15]. Kim *et al *investigated 195 Korean subjects and found no difference between β*2*-adrenergic receptor genotypes (Arg16Gly and Gln27Glu) in relation to plasma glucose, fasting serum insulin, LDL, HDL, or triglyceride levels. However, in another study of 19 healthy individuals with propanolol-induced dyslipidemia, a significantly decreased HDL serum level was found in subjects homozyous for Gln27 homozygotes, and raised triglyceride levels in subjects homozygous for Glu27 [16].

The genetic role of the β*2*-adrenergic receptor gene in the development of hypertension has been the subject of many population-based studies. The distal segment of chromosome 5 (5q31.1-qtr), which harbors the β*2*-adrenergic receptor gene, has been linked both to systolic and to post-exercise diastolic BP in genome-wide scans performed in large study populations [17,18]. In a large sample of 638 individuals from 212 Polish pedigrees, maximum linkage to essential hypertension and adjusted systolic and diastolic BP was found for a marker in close proximity to the β*2*-adrenergic receptor gene. However, no significant association of the Arg16Gly and Gln27Glu polymorphisms was detected with these parameters in the same study [19].

The published studies that have shown an influence of these polymorphisms on BP and hypertension are confusing. On the one hand, the Gly16 and Glu27 genotypes were significantly associated with the prevalence of hypertension and systolic BP in two large populations of Chinese individuals [20,21]. On the other hand, a more than twofold increased relative risk for hypertension and higher BP was detected in homozygous Arg16 carriers with diabetes [22]. One could argue that this represents a linkage phenomenon in two different populations, but in view of the functional relevance of these polymorphisms, such contradictory results remain difficult to explain. Finally, in the most extensive and prospective population based study, the Cardiovascular Health Study, with over 5000 participants, no effect of the Arg16Gly and Gln27Glu polymorphisms on BP control, new-onset hypertension, ankle-arm index, carotide intima-media thickness, or brachial flow-mediated dilation was detected [23]. Interestingly, in this study cohort, Glu27 carriers (heterozygous and homozygous) had a significantly lower risk for coronary events, and there was a similar trend for Gly16 carriers [24]. Recently, in the same study population, it was shown that Glu27 carriers also have a significantly lower risk for sudden cardiac death [25].

In contrast to the aforementioned studies, we investigated a cohort of patients in which an OSA-induced, elevated sympathetic activity exacerbated BP, heart rate, and HDL levels. Thus, the known functional relevance of these polymorphisms *in vitro *would be likely to be detected *in vivo *in such patients. The fact that we could not detect any significant result underlines the minor effect of β*2*-adrenergic receptor polymorphisms in the development of these cardiovascular risk factors, and supports the results of the Cardiovascular Health Study.

Sympathetic activation also influences the risk of MI [26]. Thus, patients with OSA show a higher incidence of fatal and non-fatal cardiovascular events [9]. In our study group, with a mean age of 56 years, we found a high percentage of CHD (12.8%) and survived MI (6.3%). Although no effect was seen on the prevalence of CHD, we found a clear and independent protective effect of the heterozygous β*2*-adrenergic receptor genotypes on survived MI. This result again supports the hypothesis that was deducted form the Cardiovascular Health Study; namely, that variations of the β*2*-adrenergic receptor gene influence vulnerability to coronary events.

In the statistical model that was chosen in the Cardiovascular Health Study, carriers of the Gly variation were pooled into one group. It is not apparent whether heterozygosity alone is protective, but a protective effect of heterozygosity in the β*2*-adrenergic receptor has already been shown in pulmonary research. Joos *et al *examined 587 smokers from the Lung Health Study. Those subjects who were heterozygous at position 27 of the β*2*-adrenergic receptor gene had a significantly lower decline in lung function at 1-year follow-up compared with the homozygous genotypes (*P *< 0.001) [27]. Our results support those findings; by pooling homozygous and heterozygous individuals into two different groups, we could show a protective effect of heterozygosity on MI, independent of other risk factors such as diabetes, arterial hypertension, dyslipidemia and BMI in a logistic regression model. Interestingly, patients heterozygous at position 27 were slightly but significantly older in spite of the lower rate of MI (Table 1). This may be an additional indicator for the protective effect of this polymorphism.

As discussed previously by Loos *et al*, [27] a reason for the protective effect of heterozygosity may be a phenomenon called heterozygous advantage. For example, a study on the role of the secretor blood group in psoriasis showed that heterozygosity had a strong protective effect (odds ratio = 0.17), even though the allele frequencies did not differ between patients and controls [28]. A possible mechanism for heterozygote advantage is interaction between the wild-type and mutant protein isoforms in heterozygous cells. This is a plausible mechanism for receptor molecules, especially in cases where they form dimers. Heterodimeric receptors consisting of a wild-type and a mutant protein isoform may have different agonist-binding, signal-transduction or agonist-promoted desensitation properties than wild-type or mutant homodimers. Several lines of evidence indicate that β*2*-adrenergic receptors form dimers like other G-protein-coupled receptors. The formation of β*2*-adrenergic receptor dimers was shown to have functional effects on receptor-stimulated adenylate cyclase activity [29]. Furthermore, the results of a study of a mutant β*2*-adrenergic receptor (Cys341Gly) suggested that the dimer is the active form of the receptor [30].

### Limitations of the study

Lipid and lipoprotein serum levels were not available for all patients. Therefore, the diagnosis of dyslipidemia had to be determined by anamnesis alone in some cases, which may have led to inaccuracies in the multivariate regression. The diagnosis of CHD was determined by anamnesis and was not controlled by coronary arteriography. Thus, our proportion of 12.8% may not reflect the real incidence of CHD.

Because our hypothesis included several risk factors for CHD and MI, Bonferroni correction after the statistical analysis should be discussed. After this correction, the results for MI would become insignificant. Because of the explorative character of our study, we refrained from performing Bonferroni corrections here.

## Conclusion

We conclude that the investigated β*2*-adrenergic receptor polymorphisms do not modulate cardiovascular risk factors in patients with OSA-induced elevated sympathetic activity. However, heterozygosity of these polymorphisms may be protective in reducing the vulnerability to survived MI, especially if heterozygosity is present for all polymorphisms. Because the statistical haplotype with heterozygosity in all three polymorphisms was not included in previous studies, we believe that this potentially protective genetic combination should be focussed on in future.

## Competing interests

The author(s) declare that they have no competing interests.

## Authors' contributions

N.K.B. and J.B. developed the study design, carried out the molecular genetic studies, participated in the statistical analysis, drafted and edited the manuscript. S.W. participated in the DNA isolation and assisted in the SSCP-analysis. J.T.E. participated in the study design and supervised the genetic research procedures, N.B., B.M.S, D.B. and L.C.R. organized the patient collection, developed the patient's queries and participated in the management of the clinical data. C.H. and A.M. participated in the statistical analysis and in editing of the manuscript.

## Pre-publication history

The pre-publication history for this paper can be accessed here:



## Figures and Tables

**Table 1:** Demographics in the different genotypes of the β2-adrenergic receptor.

Genotype	n* (% of all)	Age mean ± SD	BMI mean ± SD	Hypertension *n *(% of n*)	Diabetes *n *(% of n*)	Dyslipidemia *n *(% of n*)	CHD *n *(% of n*)	MI *n *(% of n*)
All	429	55.2 ± 10.7	31.1 ± 5.6	258 (60.1)	74 (17.2)	211 (49.2)	55 (12.8)	27 (6.3)
Codon -47								
Cys/Cys	131 (24.0)	55.9 ± 11.3	30.8 ± 5.2	76 (58.0)	23 (17.6)	65 (49.6)	16 (12.2)	11 (8.4)
Cys/Arg	195 (45.2)	55.1 ± 10.7	31.1 ± 6.0	119 (61.0)	36 (18.5)	91 (46.7)	25 (12.8)	6 (3.1)
Arg/Arg	103 (30.5)	54.2 ± 9.7	31.5 ± 5.6	63 (61.2)	15 (14.6)	55 (53.4)	14 (13.6)	10 (9.7)
*P*		0.512	0.705	0.837	0.694	0.539	0.952	**0.040**
Codon 16								
Arg/Arg	59 (13.8)	57.0 ± 9.9	31.6 ± 6.0	34 (57.6)	12 (20.8)	30 (50.8)	6 (10.9)	6 (10.2)
Arg/Gly	197 (45.9)	54.0 ± 11.1	31.1 ± 6.0	121 (61.4)	35 (17.8)	95 (48.2)	23 (11.7)	7 (3.6)
Gly/Gly	173 (40.3)	54.7 ± 10.5	31.0 ± 5.0	103 (59.5)	27 (15.6)	86 (49.7)	26 (15.0)	14 (8.1)
*P*		0.347	0.790	0.854	0.684	0.924	0.507	**0.084**
Codon 27								
Gln/Gln	109 (25.4)	55.9 ± 11.0	31.5 ± 5.4	67 (61.5)	23 (21.1)	60 (55.0)	16 (14.7)	13 (11.9)
Gln/Glu	202 (47.1)	56.0 ± 10.5	31.0 ± 6.0	117 (57.9)	33 (16.3)	88 (43.6)	24 (11.9)	7 (3.5)
Glu/Glu	118 (27.5)	52.9 ± 10.5	30-9 ± 5.2	74 (62.7)	18 (15.3)	63 (53.4)	15 (12.7)	7 (5.9)
*P*		**0.031**	0.746	0.663	0.454	**0.087**	0.391	**0.013**

Demographics of the study group (*n *= 429) and its subgroups with the different genotypes of the β*2*-adrenergic receptor polymorphisms.

Statistical tests that revealed *P*-values below 0.1 are in **bold**. There were significantly lower rates for MI in heterozygous genotypes of Cys-47Arg and the Gln27Glu polymorphisms in spite of the significant older age in the Gln27Glu variant. In addition, there wass also a trend for a lower rate of MI in the heterozygous genotype of the Arg16Gly variation.

BMI, body mass index; CHD, coronary heart disease; MI, myocardial infarction.

**Table 2:** Blood pressure, heart rate and lipid levels in different genotypes of the β*2*-adrenergic receptor polymorphisms.

	*n*	SBP mmHg	DBP mmHg	HR beats/min	*n*	Cholesterol mg/dl	Triglycerides mg/dl	*n*	HDL mg/dL	LDL mg/dL
All	429	130.5 ± 15.7	79.6 ± 9.2	76.8 ± 9.5	360	224.7 ± 39.4	171.5 ± 95.7	278	46.8 ± 14.3	146.6 ± 35.9
Codon -47										
Cys/Cys	131	130 ± 1 ± 16.3	79.8 ± 9.4	77.0 ± 9.1	112	226.0 ± 40.3	168.6 ± 76.5	93	46.0 ± 14.3	148.9 ± 32.9
Cys/Arg	195	130.9 ± 15.6	79.6 ± 9.3	76.6 ± 8.9	163	222.5 ± 38.5	175.3 ± 102.5	123	47.3 ± 13.9	144.0 ± 38.6
Arg/Arg	103	130.1 ± 15.1	79.4 ± 8.7	76.9 ± 11.0	85	227.6 ± 40.1	167.9 ± 105.2	62	46.9 ± 15.5	148.3 ± 35.1
*P*		0.878	0.942	0.924		0.594	0.789		0.796	0.578
Codon 16										
Arg/Arg	59	133.6 ± 17.2	80.9 ± 9.3	78.4 ± 9.3	53	228.2 ± 45.7	178.5 ± 114.2	39	49.7 ± 15.9	149.5 ± 34.8
Arg/Gly	197	130.0 ± 15.9	79.8 ± 9.5	76.6 ± 9.4	158	221.4 ± 38.6	164.7 ± 92.5	123	47.0 ± 14.4	143 ± 37.2
Gly/Gly	173	129.9 ± 14.9	79.0 ± 8.9	76.5 ± 9.7	149	227.2 ± 37.8	176.0 ± 91.8	116	45.7 ± 13.8	149.4 ± 34.9
*P*		0.268	0.389	0.371		0.345	0.496		0.305	0.348
Codon 27										
Gln/Gln	109	130.7 ± 14.5	80.2 ± 9.0	77.2 ± 9.2	93	222.7 ± 42.3	175.9 ± 89.4	74	44.9 ± 14.0	145.6 ± 32.9
Gln/Glu	202	130.8 ± 15.2	79.2 ± 9.1	76.6 ± 9.2	164	225.3 ± 38.3	174.1 ± 100.9	128	47.2 ± 14.3	146.5 ± 38.2
Glu/Glu	118	130.5 ± 15.7	79.9 ± 9.5	76.8 ± 10.2	103	224.8 ± 39.4	163.3 ± 93.0	76	48.0 ± 14.8	147.8 ± 35.2
*P*		0.840	0.592	0.859		0.841	0.585		0.395	0.930

Comparison of blood pressure, heart rate, lipid and lipoprotein values between the different genotypes of the β*2*-adrenergic receptor polymorphisms in untreated patients with OSA. Total cholesterol and triglyceride levels were available for 360 patients and lipid electrophoresis for 278 patients. There were no significant differences between the genotypes.

SBP, systolic blood pressure; DBP, diastolic blood pressure; HDL, high-density lipoprotein; HR, heart rate; LDL, low-density lipoprotein.

**Table 3:** Risk factors, coronary heart disease and myocardial infarction in haplotypes of the β*2*-adrenergic receptor.

	All					
		Haplotype 1	Haplotype 2	Haplotype 3	Haplotype 4	Haplotype 5
		
*n *(%)	429	113 (26.3)	78 (18.2)	47 (11.0)	42 (9.8)	34 (7.9)
Diabetes, *n *(% of Hap.)	74 (17.2)	21 (18.6)	12 (15.4)	7 (14.9)	10 (23.8)	5 (14.7)
Hypertension,, *n *(% of pt)	258 (60.1)	70 (61.9)	48 (61.5)	25 (53.2)	24 (57.1)	21 (61.8)
Dyslipidemia *n *(% of Hap.)	211 (49.2)	49 (43.4)	38 (48.7)	21 (44.7)	22 (52.4)	16 (47.1)
CHD, *n *(% of Hap.)	55 (12.8)	14 (12.4)	9 (11.5)	7 (14.9)	3 (7.1)	4 (11.8)
MI, *n *(% of Hap.)	27 (6.3)	2 (1.8)	6 (7.7)	3 (6.4)	3 (7.1)	4 (11.8)
		***P *= 0.021**				

• Haplotype 1: T/C at position -47, A/G at position 16, C/G at position 27.

• haplotype 2: C/C at position -47, G/G at position 16, G/G at position 27.

• haplotype 3: T/C at position -47, G/G at position 16, C/G at position 27.

• haplotype 4: T/T at position -47, A/G at position 16, C/C at position 27.

• haplotype 5: T/T at position -47, A/A at position 16, C/C at position 27.

Distribution of cardiovascular risk factors, CHD and MI in the five most frequent statistical haplotypes of the three β*2*-adrenergic receptor polymorphisms. Comparisons between the haplotypes were performed with the χ^2 ^test. Haplotype 1, with heterozygosity in all three polymorphisms, showed a significant (*P *= 0.021) lower rate of MI [*n *= 113, MI = 2 (1.8%]) compared with the rest of the study group [*n *= 316, MI = 25 (7.9%)]. This corresponds to an odds ratio of 0.21. There were no additional significant relations between clinical diagnoses and the β*2*-adrenergic receptor haplotypes. Furthermore, no significant differences in blood pressure, heart rate or lipid levels, respectively, existed between the different haplotypes (results not shown). Significant *P *factor is shown in **bold**.

CHD, coronary heart disease; Hap., haplotype; MI, myocardial infarction.

**Table 4:** Relative risk for myocardial infarction in OSA patients heterozygous for β*2*-adrenergic receptor polymorphisms.

	*n *(%)	Relative risk for myocardial infarction: odds ratio (95% confidence interval)	*P*
All	429		
Heterozygote: Cys-47Arg	195 (45.2)	0.32 (0.13–0.82)	**0.012**
Heterozygote: Arg16Gly	197 (45.9)	0.39 (0.16–0.94)	**0.031**
Heterozygote: Gln27Glu	202 (47.1)	0.37 (0.15–0.89)	**0.023**
Haplotype Cys-47Arg/Arg16Gly/Gln27Glu	113 (26.3)	0.21 (0.05–0.90)	**0.023**

Significant *P *factors are shown in **bold**.
